# Evolutionary Pattern Comparisons of the SARS-CoV-2 Delta Variant in Countries/Regions with High and Low Vaccine Coverage

**DOI:** 10.3390/v14102296

**Published:** 2022-10-19

**Authors:** Jiahao Zhang, Linqian Fan, Hanli Xu, Yuanhui Fu, Xianglei Peng, Yanpeng Zheng, Jiemei Yu, Jinsheng He

**Affiliations:** College of Life Sciences and Bioengineering, Beijing Jiaotong University, Beijing 100044, China

**Keywords:** SARS-CoV-2, vaccine coverage, genetic divergence, co-mutation, immune pressure, methylation

## Abstract

It has been argued that vaccine-breakthrough infections of SARS-CoV-2 would likely accelerate the emergence of novel variants with immune evasion. This study explored the evolutionary patterns of the Delta variant in countries/regions with relatively high and low vaccine coverage based on large-scale sequences. Our results showed that (i) the sequences were grouped into two clusters (L and R); the R cluster was dominant, its proportion increased over time and was higher in the high-vaccine-coverage areas; (ii) genetic diversities in the countries/regions with low vaccine coverage were higher than those in the ones with high vaccine coverage; (iii) unique mutations and co-mutations were detected in different countries/regions; in particular, common co-mutations were exhibited in highly occurring frequencies in the areas with high vaccine coverage and presented in increasing frequencies over time in the areas with low vaccine coverage; (iv) five sites on the S protein were under strong positive selection in different countries/regions, with three in non-C to U sites (I95T, G142D and T950N), and the occurring frequencies of I95T in high vaccine coverage areas were higher, while G142D and T950N were potentially immune-pressure-selected sites; and (v) mutation at the N^6^-methyladenosine site 4 on ORF7a (C27527T, P45L) was detected and might be caused by immune pressure. Our study suggested that certain variation differences existed between countries/regions with high and low vaccine coverage, but they were not likely caused by host immune pressure. We inferred that no extra immune pressures on SARS-CoV-2 were generated with high vaccine coverage, and we suggest promoting and strengthening the uptake of the COVID-19 vaccine worldwide, especially in less developed areas.

## 1. Author Summary

Tremendous efforts have been invested in battling human coronavirus disease 2019 (COVID-19) caused by SARS-CoV-2, among which a vaccine was the most plausible intervention. However, it was argued that vaccine-breakthrough infections of SARS-CoV-2 would likely accelerate the emergence of novel variants due to immune evasion. Our study explored the evolutionary directions and mutation patterns of the Delta variant in the countries/regions with relatively high and low vaccine coverage and found that low vaccine coverage areas (Africa and India) presented more mutations than high vaccine coverage areas (Israel, Singapore and the USA), which may promote the ongoing emergence of variants of concern. Several mutations, such as G142D and T950N, may exhibit a selective advantage for viral fitness. As with the increase in natural infection, the mutation frequencies of these sites increased in the low vaccine coverage areas. Though evolutionary differences existed in the countries/regions with high and low vaccine coverage, we speculated that vaccination may not place extra immune pressure on SARS-CoV-2. We suggest promoting and strengthening uptake of the COVID-19 vaccine worldwide, especially in less developed areas.

## 2. Introduction

Since its emergence in late 2019, the coronavirus disease 2019 (COVID-19) pandemic caused by SARS-CoV-2 has become a significant concern for public health worldwide. The pandemic has had a devastating impact on society and forced a global economic recession. As of mid-March 2022, more than 460 million confirmed cases have been reported, including 6 million deaths (https://covid19.who.int, accessed on 14 March 2022). Tremendous efforts have been made and multiple vaccines have been developed against SARS-CoV-2 (https://www.covid-19vaccinetracker.org, accessed on 14 March 2022). Though great success in reducing hospitalization, severe cases and deaths have been achieved from the vaccination campaign [1,2,3], the spread of the virus has not been stopped around the world, and it is commonplace to hear of coronavirus cases rebounding whenever and wherever the COVID-19 mitigation measures are lifted. Moreover, highly transmissible variants have emerged, and vaccine-breakthrough infections have also been reported [4,5,6,7].

SARS-CoV-2 is a virus with large genome, about 30 kb in length, and has been reported to accumulate two mutations per month in the global population [8,9]. Until now, the mutations have been mostly neutral or mildly deleterious and caused no changes in SARS-CoV-2 biologically, but some mutations may exhibit a selective advantage for the viral fitness, which may alter the infectivity, transmissibility and antigenicity of the virus phenotype. For example, the spike protein with a single amino acid substitution D614G conferred a moderate advantage for infectivity and transmissibility [10,11], N439K was associated with enhanced receptor-binding affinity and conferred resistance to several monoclonal antibodies [12], while mutations in the Omicron variant showed increased household transmission and immune evasion compared with previous variants [13,14]. Currently, with the spread of the virus and large-scale vaccination, a growing proportion of the population has produced natural-infected and vaccine-induced immunity against the virus [15], which inevitably confers extensive selective pressure on the virus and perhaps may lead to the emergence of antibody-escape mutants.

The implementation of measures to combat SARS-CoV-2, including non-pharmaceutical interventions and vaccination, varied greatly in different countries/regions. Vaccine development and production capacity, as well as distribution plans, are challenges for applying vaccines to the population. Israel was a world leader in vaccinating against COVID-19, and its early vaccination campaign resulted in over half of the Israeli population being delivered two doses by April 2021 [16]. Singapore has the highest vaccine coverage in the Asia-Pacific region; as of 31 July 2021, over 60% of people had completed the initial vaccination protocol. In the USA, different types of COVID-19 vaccines have been developed and authorized; as of the end of June 2021, nearly 50% of the population has completed the vaccination protocol. India is the world’s biggest vaccine producer, but it has a very low vaccine coverage; as of the end of June 2021, only 4% of Indians are fully vaccinated, and even by mid-November 2021, only 26% of the population has been fully vaccinated. Africa has a frightfully low vaccine coverage, the lowest around the continents; by mid-November 2021, the coverage rate in most African countries was still less than 10% (https://ourworldindata.org/covid-vaccinations, accessed on 15 November 2021). The countries/regions selected in this study should have enough SARS-CoV-2 sequences with high quality, and by the end of June 2021, the countries/regions that had vaccine coverage of about 50% were designated as highly vaccinated, while the ones had vaccine coverage of less than 5% were defined as low-vaccinated. Although the SARS-CoV-2 sequences in African countries were of low quantity and quality, since the Alpha and Omicron variants originated from there, this region played an important role in the SARS-CoV-2 viral evolution, therefore the whole African region was included, and the resulting deviation was discussed. Here, the USA, Singapore and Israel were representative countries/regions with high vaccine coverage, while Africa and India were representative countries/regions with low vaccine coverage. It was argued that breakthrough infections may speed up the emergence of new variants that potentially escape host immunity [17]. To explore if there was any difference existing in the evolutionary patterns between the countries/regions with high and low vaccine coverage, we used the SARS-CoV-2 Delta variant as a model variant to carry out relevant analysis in this study.

The Delta variant was first detected in October 2020 in India and became the predominant variant globally in 2021. Compared with the Alpha variant, the Delta variant was more transmissible, and carried a higher risk of hospitalization according to the preliminary clinical data [18,19]. Studying the mutagenesis mechanisms of the Delta variant is beneficial for understanding viral transmission and evolution. Our previous work tracked and monitored the genetic variations of its early sequences (before July 2021) [20]. In this study, we collected the Delta variant sequences in five different countries/regions with relatively high or low vaccine coverage, from July 2021 to December 2021, and analyzed the single nucleotide polymorphism (SNP) and co-mutation distributions to explore the viral evolutionary patterns in the countries/regions with high and low vaccine coverage. The results suggested that although certain differences existed between countries/regions with high and low vaccine coverage, no evidence supports that the differences were caused by host immune pressure. It is important to promote and strengthen vaccination worldwide, especially in less developed areas.

## 3. Materials and Methods

### 3.1. Sequence Retrieval and Selection

All the SARS-CoV-2 genomic sequences in this study were retrieved from the Global Initiative on Sharing All Influenza Data (GISAID, Munich, Germany, https://www.gisaid.org) by selecting the “VOC Delta GK (B.1.617.2 + AY.*)” as the “Variants” search term. The sequences were randomly selected and downloaded, ensuring that 10 sequences were collected from the same day and the same country/region. The low-quality (with gaps or containing “Ns”) sequences for the full-length genome and the S gene were removed. The genomic sequence of the prototype SARS-CoV-2 (NC_045512.2) was downloaded from the National Center for Biotechnology Information (NCBI, Bethesda, MD, USA) GenBank Database (http://www.ncbi.nlm.nih.gov/genbank, 249: accessed on 15 April 2022).

### 3.2. Sequence Alignment and Data Set Clustering

All the downloaded sequences were annotated by the accession ID, collection date and location, and were aligned with the multiple sequence alignment program (MAFFT, http://mafft.cbrc.jp/alignement/server/, version 7). The nucleotide pairwise distances between all the sequences were calculated by MEGA software (version 7.0.26) with the Kimura 2-parameter model, and the distance matrix was inputted into a multi-dimensional scaling (MDS) algorithm from the R cmdscale package (R version 4.1.2).

### 3.3. Root-to-Tip Divergence

The divergence pattern and clock-likeness of the full genomic nucleotide sequences of SARS-CoV-2 were plotted by root-to-tip divergence against sampling time using TempEst (version 1.5.3, formerly called Path-P-Gen) with phylogenetic trees [21]. The trees were constructed using IQTREE (version 2.1.3) under a general time-reversible (GTR) model with a discrete gamma distribution to model inter-site rate variation. The earliest Delta sequence and the first sequence for each country/region with high quality were included to calibrate the molecular clock applied for the analysis.

### 3.4. Shannon Entropy Calculation

The nucleotide and amino acid sequence diversity of the spike (S) gene for each location was calculated as site-by-site Shannon entropy values using the Entropy-One Tool (https://www.hiv.lanl.gov/content/sequence/ENTROPY/entropy_one.html, accessed on 24 February 2020).

### 3.5. SNP Calling and Co-Mutation Analysis

R codes for SNP calling and co-mutation analysis were compiled and were deposited to Github with accession number DOI. 10.5281/zenodo.7077349. The early Delta variant sequence (EPI_ISL_1360317) and the SARS-CoV-2 prototype (NC_045512.2) were, respectively, used as a reference sequence. Data were analyzed with GraphPad Prism (version 8.0.2).

### 3.6. Selective Pressure Analysis

Statistically supported sites with positive selection were analyzed through the Hyphy package. The mixed-effects model of evolution (MEME), fast unbiased Bayesian approximation (FUBAR), and sing-likelihood ancestor counting (SLAC) were used for the analysis. After merging the results from the three models, we designated the sites supported by all three methods as strongly selected sites.

### 3.7. Structure-Based Affinity Prediction

To characterize the impact of mutations on the receptor-binding domain (RBD), the interactions of the RBD of the SARS-CoV-2 variants with the human angiotensin-converting enzyme 2 (hACE2) receptor in host cells were examined using the HDOCK server (http://hdock.phys.hust.edu.cn/, accessed on 13 November 2021), based on a hybrid algorithm of template modeling with default parameters. The predicted structures were further visualized and annotated by PyMOL software (version 2).

## 4. Results

### 4.1. Sequence Information

Due to the small differences in vaccine coverage among countries/regions in the early epidemic period of the Delta variant, the sequences selected in this study were collected from 1 July 2021 to 31 December 2021. A total of 1568, 1879, 1959, 1744 and 1703 nearly full-length sequences (29,409 bp) were, respectively, downloaded from Africa, India, the USA, Singapore and Israel. After removing the low-quality sequences with gaps and Ns, there were 430, 243, 565, 708 and 414 full-length sequences and 809, 423, 819, 1479 and 1180S gene sequences left in each country/region. Singapore had the most high-quality sequences, followed by Israel, the USA and Africa, and India had the least (Figure S1A). In terms of time distribution, each month harbored a relatively balanced number of sequences in different countries/regions, except for December in Africa, October in India and August in the USA (Figure S1B).

### 4.2. The Sequences Were Grouped into Two Large Clusters and the R Cluster Was Dominant

**Figure 1:**
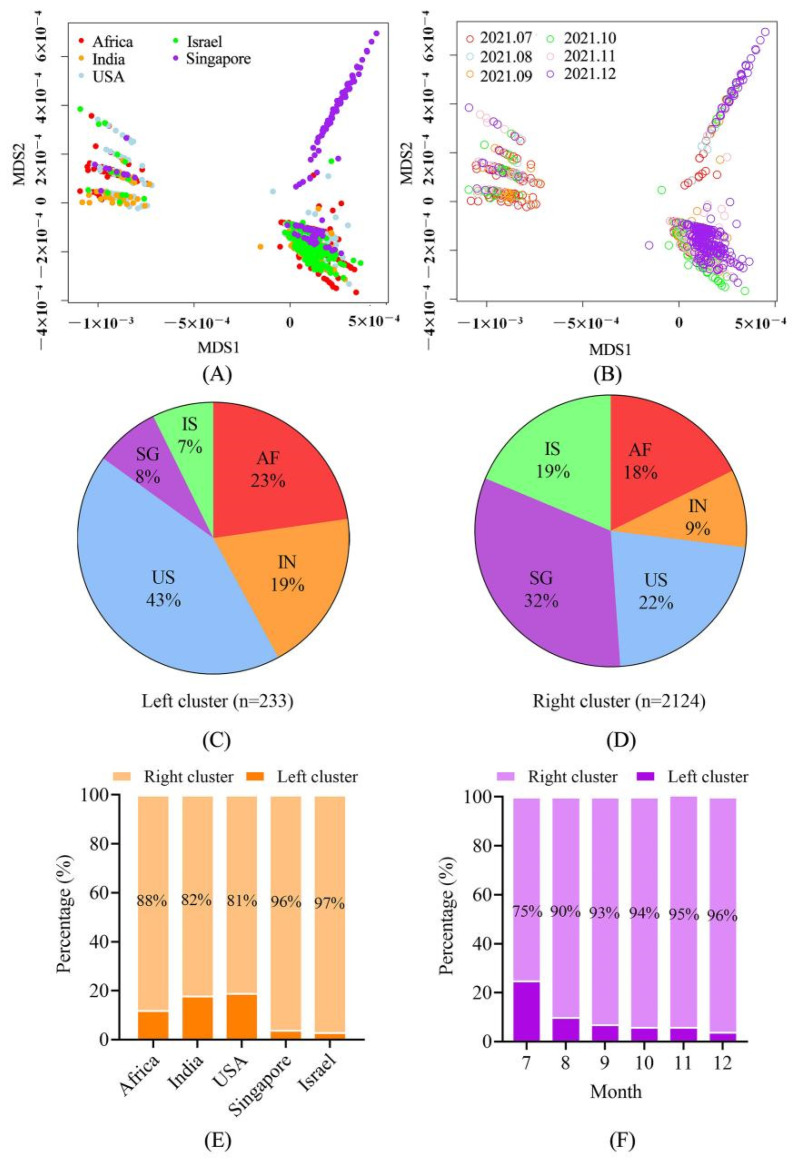
Genetic signatures and spatial-temporal distribution of the sequences. (**A**,**B**) Sequence clustering by multidimensional scaling (MDS) analysis. The sequences were grouped into R and L clusters, and the two clusters contained the sequences collected in different areas across different months. (**C**–**E**) Geographical distribution of the sequences in R and L clusters. Israel and Singapore contained much larger proportions of the R cluster than other countries. (**F**) Temporal distribution of the sequences in R and L clusters. The proportion of the L cluster decreased over time. AF, Africa; In, India; US, USA; SG, Singapore; IS, Israel.

To explore the overall genetic signature for the sequences, multidimensional scaling (MDS) plots were constructed based on the nucleotide distance matrix of the full-genome sequences. Results showed that the sequences were grouped into two large clusters: Left (L) and Right (R). Further analysis revealed that there were 12 nucleotide differences between the R and L clusters: G4181T, C5184T, C6402T, C7124T, C8986T, G9053T, C10029T, G11201A, A11332G, C19220T, C27874T and G28916T, among which C5184T and G11201A were located in the sequences of the L cluster and the other ten sites were all located in the R cluster. R contained many more sequences than the L cluster; 2124 versus 233. The two clusters contained the sequences collected in different areas across different months (Figure 1A,B). Geographical distribution analysis showed that in the L cluster, the USA accounted for the largest proportion (43%), followed by Africa and India (23% and 19%), and Singapore and Israel had the least (7% and 8%) (Figure 1C); while in the R cluster, Singapore had the largest proportion (32%), India had the least (9%), and the other three countries were comparable (18%, 19% and 22%) (Figure 1D). In total, Israel and Singapore contained the least proportion of the L cluster (Figure 1E). It is worth mentioning that the numbers of sequences from Singapore that commonly harbored two additional mutations (C5497T and C12100T) in the R cluster were not tightly clustered together with other sequences. In terms of time distribution, it was shown that the proportion of the L cluster decreased over time, with the highest proportion of 25% in July and the lowest of 4% in December (Figure 1F).

### 4.3. Low Vaccinated Countries/Regions Sequences Exhibited High Genetic Divergence

**Figure 2:**
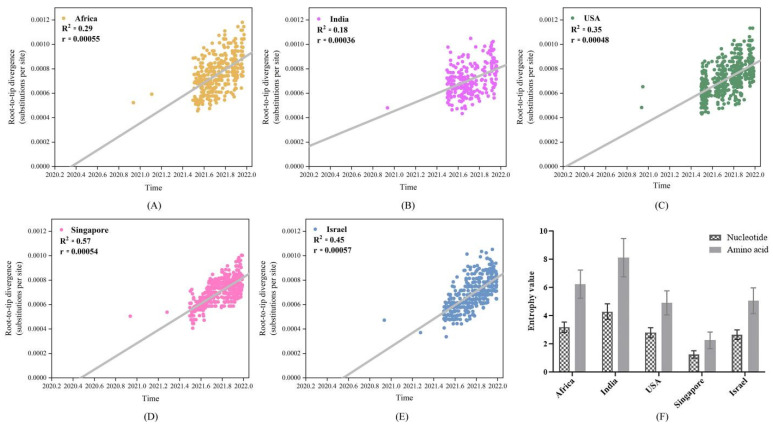
Genetic divergence plot of the SARS-CoV-2 sequences in different countries/regions. (**A**–**E**) Root-to-tip regression analysis. The phylogeny of Singaporean sequences exhibited the strongest association, followed by Israel and the USA, while the African and Indian sequences showed more diffuse regression plots. (**F**). Genetic variability of the Spike (S) sequences. The S sequences from India and Singapore, respectively, presented the highest and the lowest diversity at both the nucleotide and amino acid levels. Bars stand for the mean value calculated from individual residues values. Standard errors are shown for each bar.

Root-to-tip linear regression analyses between genetic divergence and sampling dates were performed using the best-fitting root. The results demonstrated that the phylogeny of Singaporean sequences exhibited the strongest association (R^2^ = 0.57), followed by Israel and the USA (R^2^ = 0.45 and 0.35, respectively). The African and Indian sequences showed more diffuse regression plots (R^2^ = 0.29 and 0.18, respectively), suggesting a minor clock-like pattern of molecular evolution. All the datasets from the five different places exhibited positive correlations and appeared to be suitable for phylogenetic molecular clock analysis. It was estimated that the whole-genome evolutionary rate of the virus was low in India, at 3.6 × 10^−4^ substitutions/site/year, while they were similar in Singapore, Israel, Africa and the USA, which were between 4.8 × 10^−4^ and 5.7 × 10^−4^ substitutions/site/year (Figure 2A–E). Moreover, the genetic variability of the encoding region of the S protein in different countries/regions was calculated using Shannon entropy. It was revealed that the S sequences from India and Singapore, respectively, presented the highest and the lowest diversity at both the nucleotide and amino acid levels (Figure 2F).

**Figure 3:**
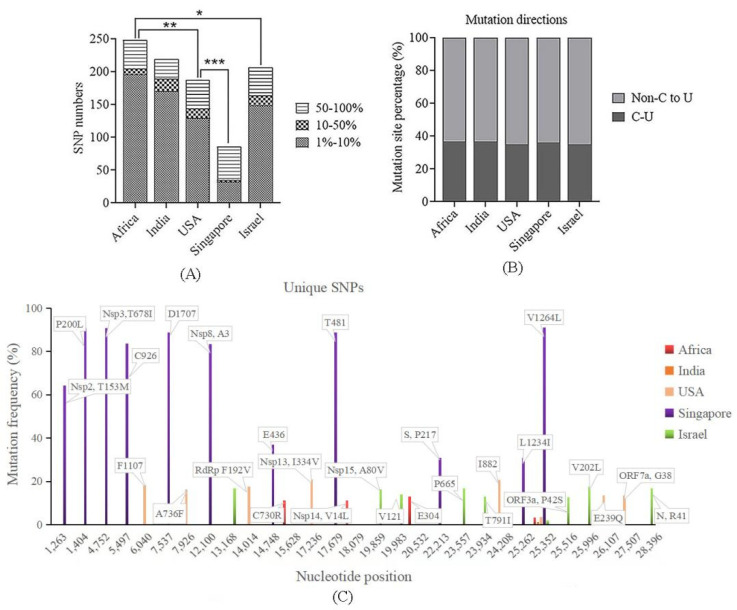
Single nucleotide polymorphism (SNP) distributions. (**A**) Numbers of SNPs in different countries/regions. Africa had the most SNPs, while Singapore had the least. *, *p* < 0.05; **, *p* < 0.01; ***, *p* < 0.001. (**B**) Mutation directions of Non-C to U and C to U. The proportion of C to U mutations in all five countries/regions was similar (around 36%). (**C**) Unique SNPs with a frequency >10% in the different areas. A total of 27 unique sites were detected, with Singapore having the largest number and the highest-occurring frequency.

SNP callings were performed to understand the genetic variations of the sequences. To avoid causing a sequencing error, a site with a mutation frequency of more than 1% was defined as an SNP. The results showed that among the five different countries/regions, Africa had the largest number of SNPs, followed by India, the USA and Israel, and among them comparable numbers of SNP were found, while Singapore had the least. Statistical analysis demonstrated that except for India, the number of SNPs in Africa was statistically different from that in the USA, Singapore and Israel (*p* < 0.05), and the number of SNPs in Singapore showed strong statistical differences from the other four countries/regions (*p* < 0.001) (Figure 3A). However, the proportion of highly occurring SNPs (≥50%) in Singapore was nearly up to 60% (51/86), much higher than those in the other four countries/regions, which led to the number of highly occurring SNPs in Singapore being comparable to those in the other places. Low-occurring SNPs accounted for a dominant proportion in Africa, India, the USA and Israel (Figure 3A). Non-C to U transitions should be of special attention as the C to U transition is a preferred direction of nucleotide mutations in SARS-CoV-2. The proportion of C to U mutations, about 36%, was observed in all five countries/regions, and a marginal difference was found among them (Figure 3B).

Unique SNPs with an occurring frequency >10% in different countries/regions were analyzed. The results showed that a total of 29 unique sites were detected, with only one site (C13168U) located in the intergenic region; all the other sites were in the coding regions, among which ten were synonymous mutations. Sequences from India had no unique SNPs, while those from Africa, the USA and Israel presented three, seven, and eight, respectively. Singaporean sequences had the largest number of unique SNPs, up to 11, and they showed much higher occurring frequencies than those in other areas, with eight sites more than 60% (Figure 3C). Further MDS plotting showed that the sequences containing one or more of these 11 SNPs were in the sub-cluster that were not tightly clustered with other sequences in the R cluster that was mentioned above (Figure S2). Among the non-synonymous mutations, two (14014-F192V and 15628-C730R) were in RNA-dependent RNA polymerase (RdRp) and one (24208-T791I) was in the S protein, and no site was detected in the RBD (Figure 3C).

### 4.4. Common Co-Mutation and Region Unique Co-Mutations Were Detected in the Countries/Regions with High and Low Vaccine Coverage

**Figure 4:**
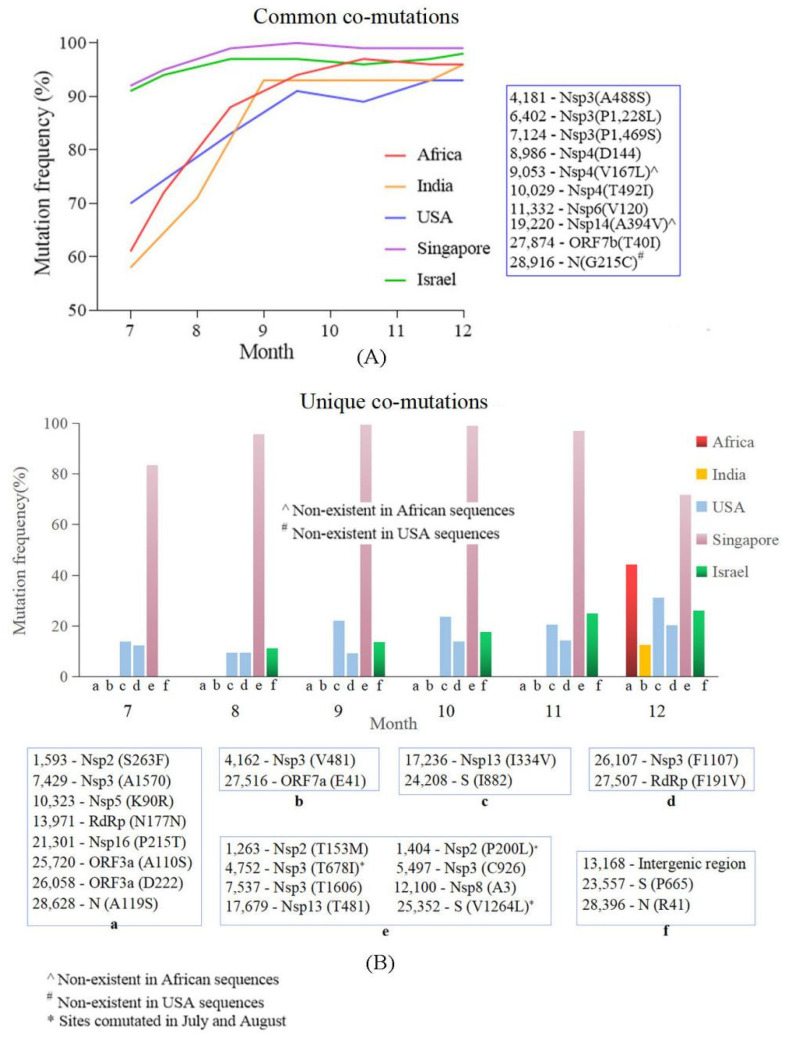
Comparisons of co-mutations patterns in different countries/regions. (**A**) Common co-mutation in the five areas. The proportion of this set of co-mutations increased over time and was extremely high in Singapore and Israel. (**B**) Unique co-mutations in different areas. Six groups (group a–f) of unique co-mutations were detected. Groups a and b were only found in December. The proportions of group c, d and e gradually increased over time. Group e was detected in Singapore throughout the six months with highly occurring frequency.

We evaluated and compared the co-mutation patterns in different countries/regions over the time course. The results showed that a set of co-mutation sites existed in all five countries/regions. Overall, the proportion of this set of co-mutations increased over time: In Africa, the proportion was 61.7% in early July, then from September, it accounted for more than 90%. In India, the proportion was 70.4% in early July, and from October, it also up to 90%. In the USA, the proportion has reached 88% since September; the proportions in Singapore and Israel have always exceeded 90%, and exceeded 95% since August (Figure 4A). This set of common co-mutations involved 10 nucleotide sites: 4181, 6402, 7124, 8986, 9053, 10,029, 11,332, 19,220, 27,874 and 28,916. Sites 9053 and 19,220 were absent in the coordinated-mutations in Africa in October. No sites were in the RBD. Notably, these ten tightly linked sites were consistent with the mutations in the sequences of the R cluster.

Meanwhile, a total of six groups (A–F) of unique co-mutations were found in different places. Groups A and B that involved eight and two sites were only detected in December in Africa and India, respectively. Groups C and D were detected in the USA all through the six months and group F was detected in Israel from August to December; the proportions of these three groups of co-mutations gradually increased over time. Group E was detected in Singapore throughout the six months with highly occurring frequencies. Interestingly, the number of co-mutated sites in group E increased over time, i.e., only three sites were involved in July (1404, 4752, and 25,352), while two new sites were added in August (7537 and 17,679) and another three were added in September (1263, 5497 and 12,100), and then, these eight sites linked tightly (Figure 4B). It is worth mentioning that all the sites in the unique co-mutations in Singapore, Israel and the USA were unique SNPs.

### 4.5. Distribution of Positively Selected Sites and Mutations in m6a Sites May Reflect Population Immunized Status

**Figure 5:**
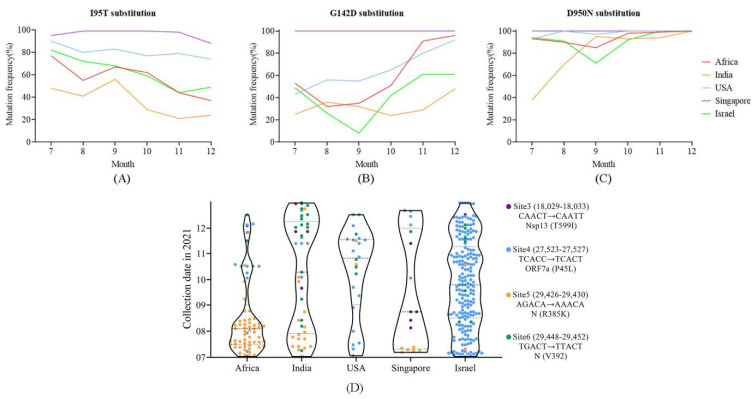
Specific mutation site analysis. (**A**–**C**) Temporal distribution of the three important amino acid sites in the S protein under strong positive selection in different countries/regions. The occurring frequencies of G142D and D950N increased over time. (**D**) Spatial-temporal distribution of the 361 sequences with mutations at m^6^A modification sites 3, 4, 5 and 6; each dot stands for a sequence. Mutations at site 4 were mainly distributed in Israel across different time, while mutations at site 5 were detected at the early stage and mainly distributed in Africa. The annotations of different sites are shown on the right side of the figure.

We examined the codons that provide strong evidence of positive selection in the S gene. The sites that were supported by all three methods (*p*-value < 0.1 in MEME and SLAC and posterior probability >0.9 in FUBAR) were defined as candidates under strong selective pressure. It was found that L5F, I95T, G142D, A222V and D950N in S were strongly positively selected sites in all five countries/regions. L5F and A222V substitutions were caused by C to U transitions and their occurring frequencies were relatively low, 0.7–1.7% and 3.0–10.3% in different countries/regions, respectively, and they exhibited no obvious time distribution features (data not shown). For I95T substitution, the occurring frequencies overall showed a slight decrease over time and varied greatly in different countries/regions; Singapore had the highest frequencies (88–99%), followed by the USA (74–90%), Israel (44–82%) and Africa (37–77%), and India was the lowest (21–56%) (Figure 5A). For G142D substitution, the occurring frequency in Singapore was 100% from July to December; while in other places, they increased over time, especially in the USA and Africa, respectively, rising from 43% to 92% and from 53% to 96% (Figure 5B). For D950N substitution, the occurring frequency in Singapore was the same as that of G142D, 100% throughout the time and they also stayed quite high in the other four countries/regions. Though it was only 38% in July in India, it increased rapidly to 95% two months later (Figure 5C). The alterations of time distribution on the occurring frequency for the strong positive selection sites in different countries/regions may reflect the difference in population immunity status.

Eight N6-methyladenosine (m^6^A) sites (site 1–8) in the SARS-CoV-2 genome with inhibiting viral infection potential were reported [22]. Mutations at the m^6^A sites, which were expected to disrupt the m^6^A, were evaluated and their distributions were plotted by R with the ggplot2 package in this study. It was found that a total of 361 sequences containing nucleotide mutations at the core motif of four m^6^A sites (site 3–6), among which 66.5% (n = 240), 21.9% (n = 79), 8.0% (n = 29) and 3.6% (n = 13) had modifications at site 4, site 5, site 6 and site 3, respectively. Site 6 was a synonymous mutation. Mutations at site 4 were mainly distributed in Israel (81.7%, n = 196), while mutations at site 5 were in Africa (67.1%, n = 53). The sequences collected from India, the USA and Singapore harbored relatively few recorded mutations, and the mutated sites scattered across different times. At the early stage, mutations at sites 4 and 5 occurred, with the USA and Israel first detecting the former, while Africa, India and Singapore detected the latter. Mutations at site 5 mainly existed in the early period (June to August), and mutations at site 4 were detected in all the countries since September (Figure 5D). Of note, mutations at site 4 were dominant and were evenly distributed in Israel throughout the period of the study.

### 4.6. Potential Enhanced Receptor Binding Ability of Substitutions in the RBD Key Site

**Figure 6:**
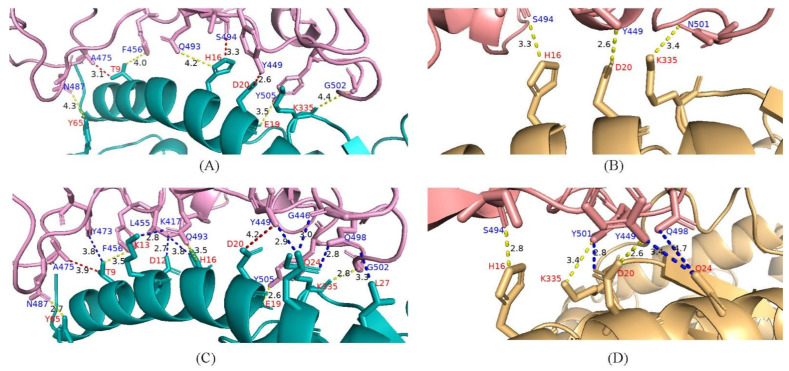
Interaction between Delta variant RBD and hACE2. (**A**,**B**) Bonding networks of L452-Q484 and N501 of Delta variant wild-type; (**C**,**D**) Bonding networks of mutants of 452R-484E and 501Y. The two mutants carrying those substitutions have potentially enhanced receptor binding capabilities of the RBD. Blue bonding stick, newly generated interactions. Red bonding stick, weakened interactions. Yellow bonding stick, increased or unchanged interactions. RBD, receptor-binding domain; hACE2, human angiotensin-converting enzyme 2.

Substitutions located on the RBD deserve more attention, as they may potentially affect the binding of the virus with the hACE2 receptor. In this study, we evaluated the docking structures between the RBD and hACE2 of the mutants carrying R452L, Q484E from Africa and N501Y from Israel. Docking energy score and root-mean square deviation (RMSD) were calculated, and the result showed that the two mutants were more stable than the early Delta virus (designated as wild-type here) (Table S1). Docking structures revealed that the amino acid substitutions significantly altered the conformation. Specifically, R452L and Q484E changed the interactions of amino acids between RBD and hACE2 as follows: (i) newly generated eight pairs of new bonding interactions (Y473-T9, K417-D12, K417-K13, K417-H16, G446-Q24, Y449-Q24, Q498-Q24 and Q498-L27); (ii) enhanced five pairs of interactions (N487-Y65, F456-T9, Q493-H16, Y505-E19 and G502-K335) and (iii) weakened two pairs of interactions (A475-T9 and Y449-D20) (Figure 6A,B). In addition, compared with the Delta wild-type, the RBD-hACE2 complex of Y501 mutants generated three pairs of new bonding interactions (Y449-Q24, Q498-Q24, and Y501-D20) and enhanced a pair of interactions (S494-H16) (Figure 6C,D). In other words, the two mutants carrying those substitutions had potentially enhanced receptor binding capabilities.

## 5. Discussion

Though the mutation rate is relatively low in coronaviruses because of the 3′-5′ exonuclease proofreading activity of the nsp14 protein [23], multiple variants of SARS-CoV-2 leading to large-scale infections globally have continuously emerged, indicating that combat with the virus will be protracted. It was revealed that the occurrence and frequency of vaccine-resistant mutations were directly proportional to vaccine coverage in Europe and America [24]. This study evaluated and compared the evolutionary patterns of the SARS-CoV-2 Delta variant based on large-scale sequences from countries/regions with high and low vaccine coverage.

Sequences involved in this study exhibited great genetic divergence and were grouped into two large clusters (L and R). The R-cluster was dominant; its proportion increased over time and overall, the high-vaccine-coverage countries (Singapore and Israel) had a higher proportion of the R-cluster than the low-vaccine-coverage ones (Africa and India). However, due to the high proportion of sequences collected in the USA that were at a very early stage (July) of this study (Figure S1), the proportion of the R-cluster was relatively low in the USA. Combined with the fact that all twelve common nucleotide differences between the L- and R-cluster were not located in the S gene, we inferred that the increase in the R-cluster was not related to neutralized antibody-resistance, in addition, it might correlate with other viral functional features associated with viral fitness, such as survival and reproductive capacity. However, we cannot rule out the possibility that it was caused by the bottleneck effect.

Genetic divergence and sampling time of the sequences in Africa and India were less congruent, indicating that only a small fraction of diverse viral populations were sequenced. There were more high-frequency SNPs in Africa than those in India, and the evolutionary rate was higher in Africa as well. This phenomenon is normal as it has been reported that two-thirds of persons with HIV infection reside in sub-Saharan Africa [25]; they are at great risk of prolonged infections and are more vulnerable to SARS-CoV-2, and more likely to harbor more quasispecies [26,27]. Nevertheless, the higher diversities in lineages in Africa could also be explained by its multiple ethnic groups and large number of countries. SARS-CoV-2 presents an extremely asymmetric mutation spectrum. Of the 12 classes of base substitutions, the C to U transition was dominant [28,29]. It usually represents a defense raised by the host cells to suppress viral infection through the APOBEC (Apolipoprotein B mRNA Editing Catalytic Polypeptide-like) family, [30,31]. In our study, the C to U was also the most common substitution and its incidence in the five different areas was similar, which were between 35–37%, reflecting no difference in mutational pressure on the SARS-CoV-2 genome in infected host cells.

Recurrent mutations can produce a phylogenetic signal that can be confused with positive selection, and it is necessary to account for this biased mutation when inferring selection [32]. We detected five sites (L5F, I95T, G142D, A222V and D950N) on the S protein with strong positive selection in all five different countries/regions, among which L5F and A222V resulted from C to U mutations and exhibited low-occurring frequencies. Spatial-temporal distributions of the other three positively selected sites (I95T, G142D and D950N) led by non-C to U mutations were evaluated, and it was found that the occurring frequencies of I95T were higher in the highly vaccinated populations. The occurring-frequencies of G142D and D950N increased over time, and we infer that they were potentially immune-pressure-selected sites, as positively selective pressure often reflects an impact of the host immune response that may further affect viral properties. Actually, the N-terminal domain (NTD) change G142D has been demonstrated to deter monoclonal antibody binding by changing the S protein conformation [33]; while D950 located in the S2 subunit is a critical site that can affect S2 refolding, and the D950N substitution showed no obvious influence on spike structure but might increase fusion ability [34].

Coordinated mutations are important markers to study viral evolution [35]; common co-mutations and region-specific co-mutations were detected in this study. The common co-mutation involving 8–10 sites (8 sites in Africa and 10 in the other four countries) presented very highly occurring frequencies in Singapore and Israel in the early stage of this study (>90%), while they exhibited relatively low frequencies in the other three countries/regions, but the frequencies increased rapidly over time and all reached 90% by December, indicating an increased immune pressure in the populations. Region-specific co-mutations in India and Africa only appeared in December, which may have resulted from increasing immune pressure from the host, but it is also possible that they were caused by the founder effect. The region-specific co-mutations in the three countries with high vaccine coverage were linked steadily all through time, with the frequencies in Israel and the USA increasing over time. This phenomenon indicated that the mutation patterns may be associated with the genetic background of the populations, or may be correlated to some feature change of the virus, e.g., infectivity, transmissibility and disease severity, just as a previous study demonstrated the possible association of co-mutations with the infection outcomes manifested in Indian patients [36]. As none of the linked sites were in the RBD, or even in the S gene, it was speculated that they may not be related to immune evasion.

The amino acid substitutions in the S-RBD would potentially influence the infectivity and pathogenicity of SARS-CoV-2. It was reported that L452R substitution can increase viral transmissibility, infectivity and resist antibody neutralization [37], while E484Q in the Kappa variant likely diminished ACE2 affinity and attenuated neutralization binding [38]. However, the results in this study suggested that the reverse mutation R452L combined with E484Q substitutions in African sequences may potentially increase ACE2 binding remarkably through changing conformation in the Delta variant. In addition, it was suggested that the N501Y substitution is an adaptive mutation that can enhance the ACE2 affinity, while having little effect on the viral neutralization activity [39,40]. Consistent with our previous study [20], this study showed that N501Y substitution in Israeli sequences had a much stronger interaction with human cellular receptors. We inferred here that R452L and E484Q in Africa may be caused by immune evasion, while N501Y may be related to enhanced viral infectivity.

RNA viruses, including SARS-CoV-2, can cap their RNAs through N7- and 2′-O-methylation to mimic host mRNAs to inhibit the innate immune response [41,42,43]. In SARS-CoV-2, a catalysis tetrad K46-D130-K170-E203 in Nsp16 was proposed to be involved in the catalytic activity of methylation [44], and Y420A substitution in Nsp 14 was reported as significantly decreasing N7-methyltransferase activity in vitro and preventing innate immune recognition [42]. Our results showed that these five sites were strictly conserved in all five countries/regions, indicating that the activity of the viral methylase had not changed. Nevertheless, recent studies revealed that RNA modification m^6^A was a novel regulator in immunity controlling, such as immune recognition, innate and adaptive immune response activation [45], and there were eight m^6^A sites in the SARS-CoV-2 genome that may potentially regulate viral infection [22]. In this study, mutations were detected at the core motif of four m^6^A sites (site 3–6). Mutations at sites 4 and 5 were predominant and were concentrated in Israel and Africa, respectively. Combined with the fact that mutations at site 5 only existed at the early stage, while mutations at site 4 had a very highly occurring frequency in Israel, and could be detected in the other four areas in the late stage, we inferred that mutations at site 4 might exhibit a stronger correlation with immune system regulation than those at other sites, if it is the viral m^6^A that inhibits SARS-CoV-2 replication. Since Israel was the first country where over 50% of the population were fully immunized, the emergence of the mutations at site 4 might be caused by immune pressure, but we still cannot rule out that it may have been caused by the bottleneck effect. The mutation in site 4 leads to P45L substitution in the ORF7a protein, and the biological significance of the substitution needs further study through functional experiments. It is worth mentioning that the T cell response has a primary focus on the antigenic sites in conserved, internal viral proteins of SARS-CoV-2 [46,47], and although the L452R and Y453F mutants were reported to escape HLA-A24-restricted cellular immunity [48], SARS-CoV-2 variants are not evolving to escape from T cell-mediated immunity [49,50].

In conclusion, this study suggested that greater genetic diversities were found in the areas with low vaccine coverage than those with high vaccine coverage, which may promote the ongoing emergence of variants of concern; though specific mutations and co-mutations with highly occurring frequencies were detected in different countries/areas, none were located in the RBD, and we could not rule out the possibility that the differences may resulted from the founder effect or other factors, such as population background. Of course, although the vaccine coverage in India and Africa was low, their natural infection rates were not, especially in India; here we cannot distinguish the difference in immunity caused by natural infection and vaccination. However, we can infer that high vaccine coverage would not cause extra immune pressure on the virus, which proved the speculation in a previous study that in SARS-CoV-2 infection, vaccine-derived immunity was unlikely to cause selective pressure for immune escape [51]. Due to the limitation of sequencing capacity and the imbalance of regional economic development, and limited areas included in this study, as well as the unclear information on vaccine type and detailed distribution, the conclusions obtained in this study may be biased. Nevertheless, we suggest promoting and strengthening vaccination through increasing COVID-19 vaccine confidence and supply in less developed countries.

## Data Availability

The data presented in this study are available in Supplemental Files.

## Supplementary Materials

The following supporting information can be downloaded at: https://www.mdpi.com/article/10.3390/v14102296/s1. Supplementary materials alongside the manuscript include files listed as follows: Figure S1, Figure S2, Supplemental table and the analyzed sequences.
