# Supplementary material for: Evolutionary Pattern Comparisons of the SARS-CoV-2 Delta Variant in Countries/Regions with High and Low Vaccine Coverage
Source: Viruses. 2022 Oct 19;14(10):2296. doi: 10.3390/v14102296 (PMC9611485; doi:10.3390/v14102296)
Supplement: Supplementary file 1 [file viruses-14-02296-s001.zip › Supplemental Table.pdf]

Table S1. Docking scores for the RBD and hACE2 complexes.

	Delta wild type	501Y mutant	452L-484E mutant
Docking score (cal/mol)	-276.54	-341.94	-302.95
Ligand RMSD(Å)	0.69	0.48	1.45

RMSD, root-mean-square deviation; RBD, receptor-binding domain; hACE2, human angiotensin-converting enzyme 2.
