# Supplementary figures and images for: Evolutionary Pattern Comparisons of the SARS-CoV-2 Delta Variant in Countries/Regions with High and Low Vaccine Coverage
Source: Viruses. 2022 Oct 19;14(10):2296. doi: 10.3390/v14102296 (PMC9611485; doi:10.3390/v14102296)

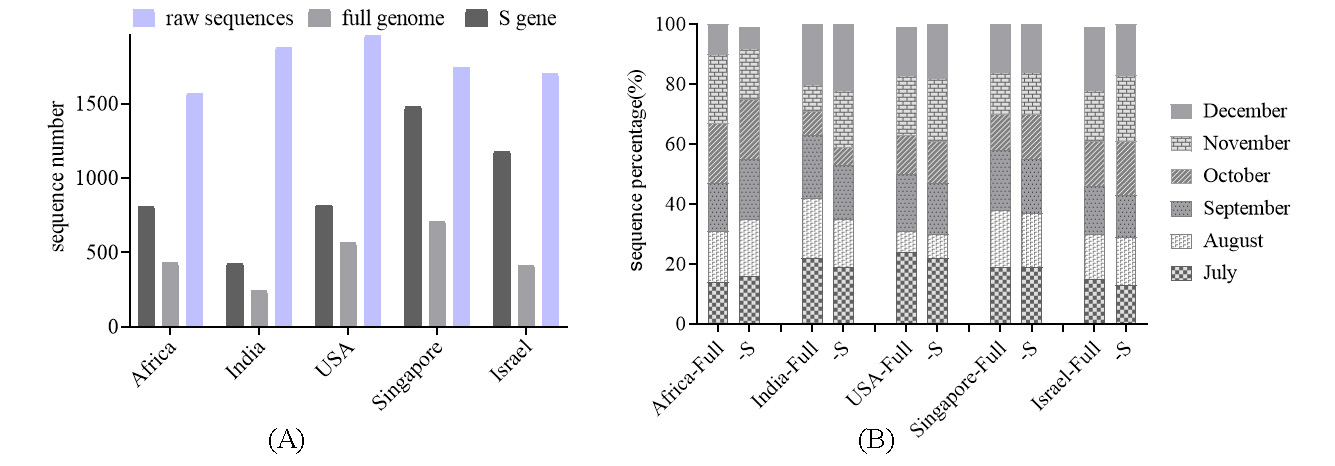

Supplement: Supplementary file 1 [file viruses-14-02296-s001.zip › Figure S1.jpg]

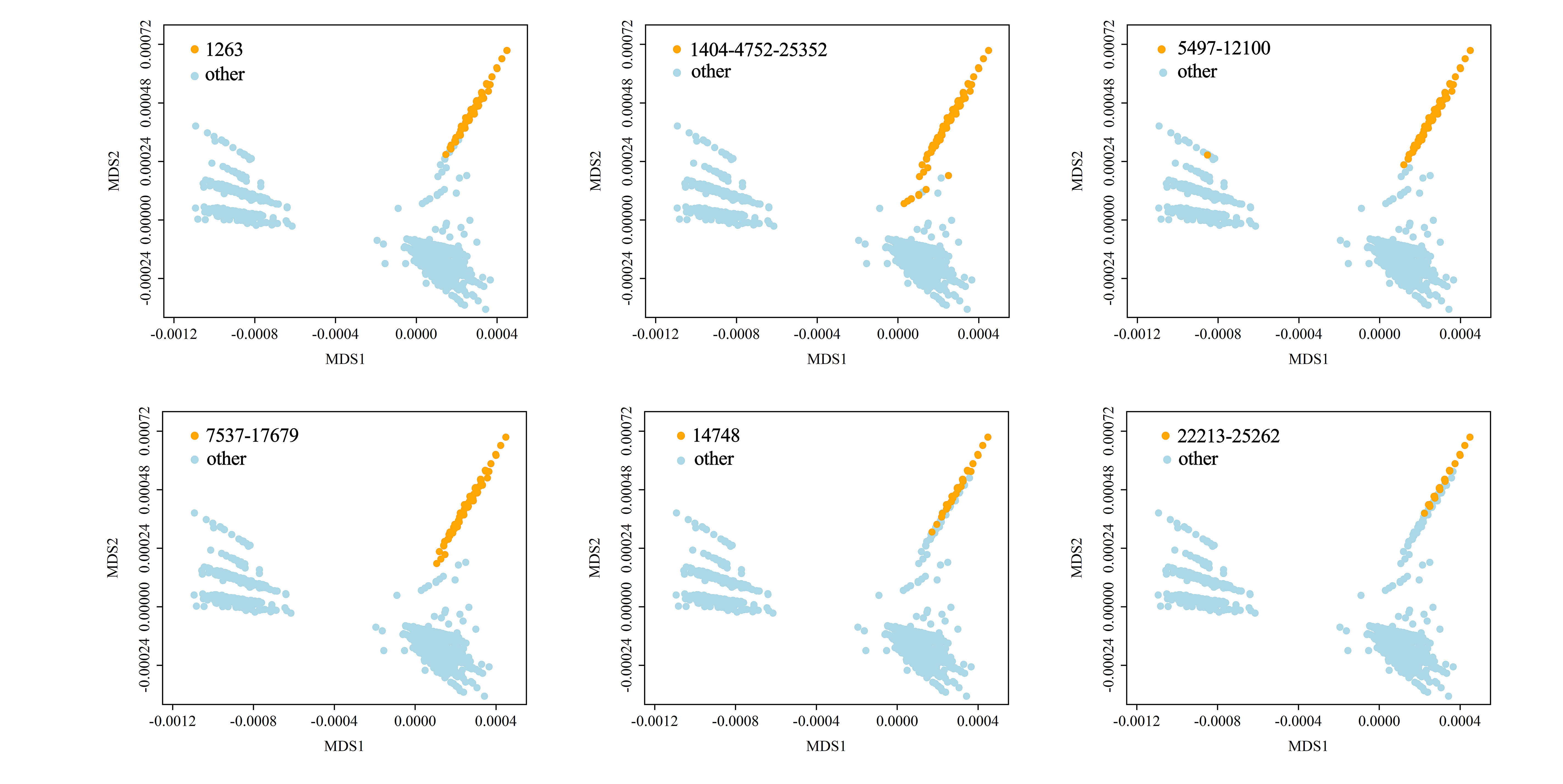

Supplement: Supplementary file 1 [file viruses-14-02296-s001.zip › Figure S2.jpg]
